# Dietary in Catarrh of the Stomach

**Published:** 1887-10

**Authors:** 


### DIETARY IN CATARRH OF THE STOMACH.

The late Dr. Thomas A. McBride, of New York City, who died very recently, on board an ocean steamer, on his return from Europe, prepared the following diet-list for use in cases of gastric catarrh:

1. Milk, cold or warm; bouillon; beef tea prepared cold, as follows: To one pound of beef cut up in pieces the size of dice, add 1 pint of distilled water and 10 drops of dilute muriatic acid. Let stand in refrigerator twenty-four hours; strain and season to taste, and if desired, warm, but not enough to make cloudy.

Peptonized milk; zwiebach, not sweetened, crackers, rusk, toast; natural Seltzer and Vichy waters, carbonated distilled water.

2. Soft boiled or raw eggs; rice or sago boiled soft in milk; clear soups; puree of potato; vermicelli or "noodle" soups; raw oysters, calves' brains, sweet breads, pigeons, chicken, calves' feet (?), the latter five articles boiled, roasted, stewed or broiled.

No vegetables, except those mentioned, to be allowed with soups. No "wheaten grits," hominy, barley or oatmeal.

3. "Minced" or finely cut boiled ham; "minced" or finely cut rare beefsteak; coffee and tea; articles under 1 and 2 as advised.

4. Rare roasted chicken, and pigeons without sauces, especially cold; venison; partridges, woodcock and snipe, not too fresh; boiled fish; white bread (stale); macaroni; baked apples; fruit jellies; a very small amount of butter, otherwise no fats at any time.

Only dry wine; no beer; no ale or porter. Rye whisky or brandy diluted with the waters mentioned may be used with lunch and dinner when pronounced necessary.—*Clinical Record.*

